# Outpatient Mental Health Nurses' Experiences of Suicide Follow‐Up Interventions: A Qualitative Interview Study

**DOI:** 10.1111/jpm.13150

**Published:** 2025-01-04

**Authors:** Sally Hultsjö, Henrika Jormfeldt, Ester Allstrin, Andreas Karlsson

**Affiliations:** ^1^ Department of Psychiatry Ryhov County Hospital Jönköping Sweden; ^2^ Division of Nursing and Reproductive Health, Department of Health, Medicine and Caring Sciences Linköping University Linköping Sweden; ^3^ School of Health and Welfare University of Halmstad Halmstad Sweden

**Keywords:** mental health nurses'experiences, qualitative research, suicide follow‐up interventions

## Abstract

**Introduction:**

Suicide is a leading cause of death worldwide. Following a suicide attempt, many patients receive suicide follow‐up interventions (SFI) from outpatient mental health care services, where outpatient mental health nurses play a crucial role. There is an urgent need to raise awareness of improvements and opportunities for development in this aspect of care to gain insights into potential areas for improvement and opportunities for development.

**Aim:**

To investigate outpatient mental health nurses' experiences of SFI.

**Method:**

A qualitative interview study was conducted with 10 outpatient mental health nurses. Conventional content analysis was used to analyse the data.

**Results:**

Three categories emerged: connecting with and understanding suicidal patients, being dependent on adequate conditions for SFI, and feeling competent but vulnerable in SFI.

**Discussion:**

Providing structured training for nurses to address patients with particularly challenging conditions is vital, as SFI entails complex and challenging situations. Training that incorporates proven methods from other interventions, involving the expertise of those with lived experience, employers, and academics, can offer significant advantages. Promoting increased collaboration can enhance the safety of assessments.

**Implications for Practice:**

Team‐based SFI can enhance suicide follow‐up intervention services in psychiatric outpatient care.

**Summary**

- What is known on the subject
  ○ Outpatient mental health nurses play a key role in assessing patient well‐being and implementing suicide follow‐up interventions over time.
  ○ Performing suicide follow‐up interventions can be challenging for outpatient mental health nurses, leading to feelings of stress and concern about the task.
- What the paper adds to existing knowledge
  ○ Outpatient mental health nurses perceive themselves as competent and capable of supporting the patient in suicide follow‐up interventions.
  ○ To enhance outpatient mental health nurses' competence and to ensure patient safety, collaboration and targeted training for patients with challenging conditions are important.
  ○ There are benefits to have training that is well‐organised and include ideas that having worked in other interventions and to involving people who have personal experience, along with employers and academics.
- Implications for practice
  ○ Professionals and carers should consider organising suicide follow‐up interventions in team‐based approaches to enhance the effectiveness of the interventions in outpatient mental health care.
  ○ Teamwork and collaborative approaches can alleviate nurses' stress and improve the quality of assessments.
  ○ Targeted training for nurses in SFI for patients with challenging conditions can enhance nurses' competence and contribute to safer suicide assessments over time.

## 1 Introduction

Suicidal ideation encompasses contemplation of self‐harm, expressed desires for self‐inflicted harm, and the formulation of concrete suicide plans (Harmer et al. 2024). There are approximately 7,00,000 suicides yearly worldwide, and for every suicide there are probably more than 20 suicide attempts (WHO 2023). Among 15–29‐year‐olds, every fourth death is caused by suicide, of which nearly 90% previously had a psychiatric diagnosis (Mehanović et al. 2023). Depressive disorder (McMahon et al. 2022), schizophrenia, bipolar disorder, anorexia nervosa, personality disorder, and younger age at the onset of suicidal behaviour are significantly associated with repeated suicide attempts (Mehanović et al. 2023). The mortality risk of suicide attempters is very high, both in the months immediately following the attempt and up to several years afterwards (Mehanović et al. 2023).

Specialised mental health care services have the principal responsibility for the detection and management of patients exhibiting suicidal ideation. Thus, after hospital discharge following a suicide attempt, 60%–70% of patients are referred to nurses in outpatient mental health care for suicide follow‐up interventions (SFI). SFI consists of supportive conversation, psychosocial or pharmacological interventions (Castaigne, Hardy, and Mouaffak 2017), brief active contact, outreach interventions such as phone calls and home visits, or digitally driven interventions (Menon and Vijayakumar 2022), or a combination of these to prevent recurrent suicide attempts.

An intervention developed over recent decades, primarily to address and mitigate self‐harming behaviours and suicide attempts as a complement to SFI, is patient‐initiated brief admission (BA) (Lindkvist et al. 2021; Liljedahl et al. 2017). This approach empowers patients by allowing them to initiate brief hospital admissions during periods of crisis, providing immediate access to professional support and a safe environment (Lindgren et al. 2024; Arnold, Wärdig, and Hultsjö 2022). However, participation assumes that BA is accessible and that the person has agreed to the intervention. Thus, BA is not available to all patients. Additionally, persons suffering from suicidal ideation are referred to outpatient SFI after a suicide attempt (Omerov et al. 2020). Thus, outpatient mental health nurses encounter persons suffering from suicidal ideation on a daily basis. A significant portion of the outpatient mental health nurses' scope of expertise and accountability pertains to suicide prevention, encompassing the identification of issues and requirements linked to patients' distress (Dahlberg, Ranheim, and Dahlberg 2016). The nurse fosters personal connections with the patient to support them in dealing with illness or suffering and to help them find meaning in their experience (Travelbee 2013).

A suicide occurs when the individual perceives their mental anguish as unmanageable, and suicide is viewed as the only way to put an end to the suffering (Pompili 2010). While it is not feasible to predict a specific suicidal act, certain factors necessitate a more thorough assessment of the individual. Factors such as feelings of hopelessness, depression, stress, and maladaptive coping mechanisms entailing guilt and distraction have predictive potential for suicidal behaviour (Rudd et al. 2006). Individuals at a heightened risk of suicide often resort to negative coping strategies, potentially due to a deficiency in awareness regarding alternative, constructive coping mechanisms (Lew et al. 2019). Moreover, severe somatic illness (McFarland et al. 2019) and self‐harming behaviours (Carroll, Metcalfe, and Gunnel 2014) are additional factors that elevate the risk of suicide.

Identification of high‐risk suicidal patients can help when planning their outpatient care following hospital discharge (Mehanović et al. 2023). Assessing suicide risk represents one of the most challenging tasks for outpatient mental health nurses, as the suicidal process involves a transformation in which life and death coexist and the individual may simultaneously plan their future while considering whether to choose death (Ovox et al. 2024). Furthermore, it has proved extremely challenging to engage patients who have attempted suicide in treatment, with between 11% and 50% declining treatment or dropping out at an early stage after treatment initiation (Castaigne, Hardy, and Mouaffak 2017).

In a previous study by Clua‐García, Casanova‐Garrigós, and Moreno‐Poyato (2021), it is highlighted that nurses experience challenges in SFI. They encounter feelings of grief, stress, restlessness, and fear, which may lead to a sense of lack of control, resulting in anxiety. Moreover, uncertainty and isolation in their work environment were noted along with difficulties in managing ethical dilemmas that may arise. The responsibility of independently assessing suicide risk can increase emotional vulnerability and evoke moral stress in nurses. Studies indicate that there are various approaches to providing SFI (Jardon et al. 2019). Intensive intervention programs provide care at home and include brief therapy sessions, while case management programs focus on maintaining contact with patients. These programs involve sending letters or postcards after a patient leaves the emergency room, providing a crisis card with a phone number for assistance and details of a place to stay if needed, and phoning the patient at some point after they have been discharged. As suicide prevention is increasingly used, further research should be conducted (Jardon et al. 2019; Castaigne, Hardy, and Mouaffak 2017). There is a need to design strategies for the assistance and care of suicide attempters in the long term in order to reduce the likelihood of further suicide attempts (Mehanović et al. 2023). From the perspective of outpatient mental health nurses, there is a need to raise awareness of SFI to gain insights into potential areas for improvement and opportunities for development in this aspect of care (Vandewalle et al. 2019).

## 2 Aim

To investigate outpatient mental health nurses' experiences of suicide follow‐up interventions.

## 3 Methods

### 3.1 Type of Research

A descriptive qualitative design was suitable as this design focuses on elucidating and narrating daily life events as experienced and articulated by the participants (Hsieh and Shannon 2005). Data was analysed using conventional content analysis. The research process regarding data collection, analysis, and compilation of results followed the COREQ guidelines (Tong, Sainsbury, and Craig 2007).

### 3.2 Participants

A purposeful sampling was employed (Polit and Beck 2021). The inclusion criteria were to have a minimum of 6 months of experience working as an outpatient mental health nurse in outpatient mental health care and experience in SFI. In total, 10 registered mental health nurses participated, of whom eight were women and two were men in the age range of 32–61 (median 43 years). Their working experience was 4.5 (median years) within outpatient mental health care.

### 3.3 Data Collection

Data collection took place from October 2023 to January 2024 in four outpatient mental health facilities in a region of southern Sweden. In the outpatient mental health teams' facilities, treatment is provided to patients with general psychiatric conditions, including depression, anxiety, eating disorders, obsessive‐compulsive disorder, post‐traumatic stress disorder, bipolar disorder, personality disorders, ADHD, and autism. The teams consist of physicians, psychologists, social workers, occupational therapists, and mental health nurses. The nurses maintain continuous contact with patients over time, while the other professionals provide time‐limited interventions based on their respective areas of expertise.

After obtaining consent from the head of the department, unit managers were contacted, who then forwarded an email with information about the study to all mental health nurses in four teams. Nurses who were interested in participating replied to the email, giving their names, contact information, and demographic data such as age, work experience, and education. To create a calm and relaxed environment, participants chose where and when the interview would take place, with all opting to conduct the interview in their offices. Two registered nurses (A.K. & E.A.) with experience in mental health care conducted the interviews. The interviews followed a semi‐structured interview guide. The initial question in each interview was: What is your experience of suicide follow‐up interventions after suicide attempts? Additional questions were, Can you describe a meeting with a patient?, and Do you experience any challenges with SFI?” In‐depth questions such as Can you tell me more about that?, “Can you describe a situation?, and How did that make you feel? were continuously added during the interviews to allow participants to elaborate on their responses and freely discuss their experiences (Hsieh and Shannon 2005). Two test interviews were conducted, and as no changes needed to be made, these interviews were included in the analysis. The interviews ranged in duration from 20 to 45 min each (median 30 min) and were audio‐recorded and transcribed verbatim.

### 3.4 Data Analysis

Conventional content analysis was utilised for data analysis as it is a suitable method to describe experiences and find similarities, differences, and patterns in the content (Hsieh and Shannon 2005). Initially, all data was read repeatedly to achieve immersion and obtain a sense of the whole. The authors immersed themselves in the data to allow new insights to emerge. Preconceived categories were avoided; instead, the categories and their names were allowed to evolve naturally from the data. Subsequently, each piece of data was examined word by word, by first highlighting the exact words from the text that appeared to capture key concepts. The highlighted key concepts were discussed and coded. The codes were sorted into 12 preliminary categories based on how they were related and linked. The preliminary categories with similar content were merged, resulting in two categories with six corresponding subcategories. Definitions for each category and subcategory were then developed, and the relationships between categories and subcategories were identified, based on their concurrence, antecedents, or consequences. To maintain the integrity of the raw data, statements were extracted and gathered with codes in categories. To avoid the analysis being modified and examined by any prior understanding, the researchers continuously consulted the raw data. Authors' personal experiences and understandings were bridled in dialogue with each other during the analysis process.

### 3.5 Ethical Responsibilities

The Regional Ethics Review Board in Linköping has approved the study, with reference number [2023‐07123‐01]. All participants received information about the voluntary nature of participation and the option to withdraw at any time without providing a reason and signed an informed consent form before entering the study (World Medical Association 2013).

## 4 Results

The results consist of three categories: Connecting to and understanding suicidal patients, Being dependent on adequate conditions for SFI, and Feeling competent but vulnerable in SFI, with associated subcategories (Table 1). The content of the categories is reinforced by quotes followed by the participant number expressing them.

**TABLE 1:** Categories and their associated subcategories of how outpatient mental health nurses' experience suicide follow‐up interventions.

Connecting to and understanding suicidal patients	Being dependent on adequate conditions for Sfi	Feeling competent but vulnerable in SFI
Building Trust with suicidal patients Connecting with patients with particularly challenging conditions	The importance of structured plans and risk assessments The importance of teamwork and collaboration	Making a difference as a nurse Feeling vulnerable in the profession

## 5 Connecting to and Understanding Suicidal Patients

In order to connect with and understand suicidal patients, nurses emphasised the importance of establishing trust with the patient, which was perceived as more difficult with certain patients who had particularly challenging conditions.

### 5.1 Building Trust With Suicidal Patients

The nurses emphasised the importance of establishing trust in the therapeutic relationship with suicidal patients. They described trust as achievable through demonstrating their availability and presence, as well as informing patients about the possibility of contacting them via phone, email, or other communication channels. It also emerged as important to clearly communicate the availability of support in case the patient's condition deteriorated acutely.

It is important to establish a strong therapeutic relationship where the patient can feel trust and dare to open up and contact the provider in case of acute deterioration. (P7)

The nurses built trust by making agreements with the patient. They described shaking hands with the patient and promising to meet again next week. Agreement between nurse and patient not to inflict any self‐harm or attempt suicide also occurred regularly.

Now I'm following up with a girl who self‐harms. During our conversation today, she may have something she doesn't want to tell me, but hopefully, she will tomorrow or next week. We still have this agreement that we will meet next week and that she will not act on her thoughts. (P8)

The nurses indicated that situations arose where the patient was not honest with the provider, which could cause the nurse's trust in the patient to deteriorate, which in turn resulted in the deteriorated quality of the therapeutic relationship.

I find it difficult when the patient has then lied to their provider and said they did not intend to end their life, but two hours later, the patient overdoses. Then the patient reveals that it was planned for a long time, and it becomes difficult when you don't know if the patient is honest. (P10)

Nurses said a significant aspect of SFI was that it revealed the underlying causes of mental health problems. A recurring theme was that nurses viewed each patient as unique and tailored interventions to address the patient's individual problems and needs. Through conversations about the attempted suicide, the nurse and patient got to know each other, which could lead to the establishment of trust and the beginning of a good therapeutic relationship.

There are so many different reasons why a patient attempts suicide; it can be severe mental illness such as depression, but it can also be due to economic or social problems, so that's one of the challenges: helping the patient find the underlying cause of why they feel so bad that they attempt suicide. (P4)

### 5.2 Connecting With Patients With Particularly Challenging Conditions

The results revealed that patients with challenging conditions presented challenges in SFI due to the complexity of their symptoms. Nurses described how patients with borderline personality disorder (BPD) often exhibited both self‐destructiveness and impulsivity. The patients' difficulties in emotional regulation often led to intense anxiety, which they attempted to alleviate through serious self‐destructive actions. These self‐destructive actions could be understood as suicide attempts, even if actual suicide was not the patient's intention. Nurses said that they partly lacked the competence to address these patients and highlighted therapeutic treatments as more helpful for the issues at hand.

Sometimes, it may not have been a suicide attempt but rather an impulsive act or a cry for help. This is often seen in patients with BPD. There may be patients whom we should not follow up in this manner; this is my reflection, as it becomes counterproductive and provides incorrect validation. (P7)

Nurses further highlighted differences in SFI across different age groups. They described how elderly patients would not usually talk openly about mental illness and tended to focus more on somatic complaints and said that their mental suffering found its outlet through somatic ailments. Younger individuals found it easier to communicate about suicidal thoughts than older individuals, who also tended to suppress their troublesome feelings and thoughts rather than open up and talk with the nurse. In turn, this contributed to a fear that elderly patients would act on their mental suffering without the nurse's knowledge.

When we look at guys born in the 1990s and onwards, they actually find it easier to talk about emotions and even express emotions in conversation. You could call it a paradigm shift. A man in his 50s or 60s never sits and cries in front of me, but if I meet a 25‐year‐old, he might actually do it and show that he is distressed. (P8)

The nurses said that patients who had difficulty opening up were challenging. The nurses stated that initially, it might be necessary to establish a connection before being able to understand who the patient was and get to know them. Nevertheless, some patients just had obvious difficulty opening up, regardless of the level of trust between the nurse and the patient and the quality of the therapeutic relationship, which caused difficulties in connecting with patients that had these challenging conditions.

I have a girl now with chronic suicidal thoughts who makes suicide plans with exact days and times. Then you walk around with butterflies in your stomach and wonder if you will see this young girl again. This is a classic example of a patient who does not want to open up, with many traumas in childhood. (P8)

## 6 Being Dependent on Adequate Conditions for SFI

In this category, nurses highlighted the conditions necessary for them to provide adequate SFI. It emerged as important and reassuring to have structured plans and assessments, and the nurses expressed a desire for more teamwork and collaboration with other healthcare providers.

### 6.1 The Importance of Structured Plans and Risk Assessments

Nurses perceived various aspects of structure in their work with SFI. Overall, they felt that there were structured plans and risk assessments to rely on. Nurses described using standardised rating scales in SFI, as well as utilising the suicide steps as a tool. They also mentioned the importance of having a care plan for each patient, containing current issues, planning, and a crisis plan. However, it emerged that not all patients had an updated care plan, although some nurses described being meticulous about writing care plans as it provides a sense of control and security. From this perspective, nurses emphasised the importance of using their clinical experience along with the assessment instruments in evaluation.

We have fixed routines according to the suicide prevention program. The region has suicide prevention officers who collect the latest findings on suicide. We have local and regional meetings. In this way, they try to align efforts to maximise the chance of equal care for all patients. I think this works satisfactorily, but it can of course be developed. (P7)

However, it also emerged that there were ambiguities in the risk assessments regarding SFI, and nurses sometimes felt uncertain. There were uncertainties about follow‐up conversations and their content. Nurses described not knowing what approach to take in the conversation or how often the patient should be followed up.

We are assigned a patient who is to be offered follow‐up for a year, and initially, we have to meet once a week, conduct structured suicide risk assessments, and have supportive conversations, as stated in the routine. But what should we talk about after a month? It's largely up to oneself; one has to come up with something. (P9)

Overall, routines were perceived as a comfort and support by nurses. While structured plans and risk assessments were important, they were secondary and not the most crucial aspect of SFI; the most important thing was to listen to the patient and have shared planning that all parties agreed on.

### 6.2 The Importance of Teamwork and Collaboration

Most nurses felt that teamwork regarding SFI worked well at the four outpatient departments, where there was openness towards each other and sensitivity to each other's workload.

… It feels like we're helping each other, and often we share a suicide prevention case. It also feels very safe for the patient because if someone is sick for a while, it doesn't all depend on that person, and above all, you can feel that some cases are complex, so it doesn't feel as heavy either. (P5)

Despite the good teamwork, several nurses described how the assessment of the patient's health and illness factors sometimes led to the need for the patient to see another professional, such as a psychologist for psychotherapy. Unfortunately, nurses felt that there could be a long wait for psychotherapy, and during this time, follow‐up conversations could feel particularly burdensome with the knowledge that the patient was in great need of therapy.

In the assessment itself, you often see which other profession may need to be involved, but it can take months before the patient gets an appointment. During this time, I do my best, but it doesn't always feel good to have supportive conversations when you know the patient is in great need of other interventions; you can feel inadequate. (P9)

Collaboration between different units was also something that participants mostly felt worked well, between inpatient and outpatient care as well as with emergency services. However, they highlighted that sometimes they felt the need for a medical assessment in case of high suicide risk. Nurses further emphasised the importance of receiving supervision in the field, which was currently available in outpatient care, though that had not always been the case. It emerged as important to receive support in SFI through both supervision and open discussions and teamwork with colleagues in the same profession and also with physicians, psychologists, and vocational therapists.

## 7 Feeling Competent but Vulnerable in SFI

Based on their holistic profession and expertise, nurses described how they felt competent and believed they could contribute to making a difference in SFI; however, they also expressed feeling vulnerable in this context.

### 7.1 Making a Difference as a Nurse

Nurses described their holistic view of the profession, seeing the whole person‐mentally, physically, and psychosocially‐as important for SFI. They emphasised understanding the patient's entire life situation and the importance of involving other professionals' expertise. Nurses believed their holistic approach could address the complex factors in SFI, such as comorbidity and psychosocial issues that impacted patients' well‐being and complicated suicide risk follow‐up. Despite challenges, they felt they could make a significant difference by understanding and supporting suicidal patients.

The nurse has a holistic view in their education, of the whole person, a holistic perspective. It is in the nurse's nature to see all aspects‐psychosocial, somatic, and psychological. All professions can be responsible for follow‐up, but in my opinion, the specialist nurse is best suited. (P7)

Nurses described how they could contribute to and support patients in various ways. By listening to the patient, the nurse could understand their needs and assess the situation, adapting conversations based on the patient's needs. When nurses connected with the patient and experienced a successful SFI, they felt that they were contributing something and making a difference.

I actually remember a lot of cases where you have been able to contribute a lot of help and support based on your professional role. There are patients where you feel that you can contribute to identifying unhealthy factors and can work, based on them, by adapting the conversations, so to speak. (P9)

### 7.2 Feeling Vulnerable in the Profession

Although nurses viewed themselves as competent for SFI, it emerged that there was a sense of vulnerability in SFI for patients with an elevated suicide risk. They said that nurses were often alone when making assessments of suicidal patients and felt isolated and vulnerable.

When a patient enters the suicide prevention program, I become solely responsible. It is a very heavy responsibility; you sit alone with a very important type of follow‐up. (P9)

It emerged as challenging to gauge the patient's thoughts regarding suicidal ideation and feelings of life‐weariness. Nurses described a feeling of being left alone with an experience of being burdened with heavy responsibility.

It can be difficult planning because you are alone in the assessments and follow‐up. What if something happens? If something does happen, so, it's a heavy responsibility to shoulder alone. (P9)

Furthermore, there was uncertainty regarding assessments in complex conversations with patients at risk of suicide. In these cases, nurses wished they had a co‐assessor to confirm that the assessment was correct, which would contribute to increased confidence and patient safety. Unfortunately, it emerged that other staff were not always available to assist, leaving the nurse reliant on their own assessment.

## 8 Discussion

The main result of this study highlights the importance of connecting to and understanding suicidal patients by establishing trust with them. This emerged as challenging in patients with particularly challenging conditions such as BPD and patients with difficulties expressing themselves. To feel confident in SFI, nurses emphasised the importance of adequate conditions for SFI, such as structured assessments and teamwork and collaboration with other professionals. Furthermore, nurses perceived themselves to be competent in SFI, although they often felt alone and vulnerable in their work.

Suicide is not predetermined and can be prevented by considerate questions about suicidal thoughts, encouraging help‐seeking behaviours, and responding to suicidal thoughts and actions (Nice 2018). In this study, nurses emphasised trust as a prerequisite in SFI and as an important factor in the therapeutic relationship between the nurse and the patient. A therapeutic relationship can help the patient shift away from suicidal thoughts towards feeling more zest for life and motivation for improvement (Vandewalle et al. 2019). Interpersonal interaction can greatly influence the patient's well‐being (Hansson et al. 2011). When trust is lacking in this interaction, it can adversely affect the outcome of the therapeutic relationship. It has been shown that trust is also crucial for nurses and patients to work together (Vandewalle et al. 2020). Nurses in this study described trust as including getting a commitment from the patient not to harm themselves. Obtaining such a commitment has also been found rewarding and relatively straightforward by nurses in other studies (Jansson and Graneheim 2018). However, this requires the nurse to be personally involved and committed. This study revealed that at times it can be challenging to engage with and to trust patients. This has also been noted among nurses in psychiatric emergency care (Helene Hem, Heggen, and Ruyter 2008). Suicidal patients are commonly perceived as difficult to trust due to ambivalence regarding their desire to live or die, and suicidal acts are often impulsive responses to acute psychosocial stressors (WHO 2014). A key component in suicide prevention is for the nurse to provide an opportunity for these patients to reflect on and talk about their suicidal thoughts and experiences (Nice 2018). To capture emotional, spiritual, and physical experiences, nurses need to tailor their communication to become more empathetic as a first step in fostering hope in the patient as a way of coping with suffering (Travelbee 2013).

In this study, nurses found it particularly challenging to understand patients with BPD as their condition was characterised by anxiety, self‐destructiveness, impulsivity, and depressive symptoms. Although all these conditions are risk factors for suicide, not all individuals exhibiting these conditions have thoughts of actually committing suicide (Leavey et al. 2017). Instead of focusing on risk factors such as depressive symptoms, the emphasis should instead be on protective factors that can be protective and avert suicide (Fedorowicz et al. 2023). On the other hand, it is dangerous to overlook the suffering of individuals with self‐destructiveness, as these patients have an approximately 33% greater risk of making further suicide attempts, especially if symptoms such as anger and outwardly aggressive behaviour are present (Mehanović et al. 2023; Stringer et al. 2013). The intensified vulnerability within the patient entails increased demands on the nurse's ability to listen to descriptions of feelings and understand the meaning and function of the patient's behaviour. According to the nurses in this study, they lacked the appropriate skills to implement SFI of patients with BPD. Studies confirm the knowledge gap among healthcare professionals when it comes to the management and treatment of individuals with BPD (Bodner, Cohen‐Fridel, and Iancu 2011; Hauck, Harrison, and Montecalvo 2013). This knowledge gap is believed to contribute to a negative attitude towards this patient group, particularly among nurses and psychiatrists (Bodner et al. 2015). It is possible to change healthcare professionals' attitudes towards patients with BPD. A relatively simple training intervention consisting of 6 h of lectures and group discussion on this patient group proved to improve the staff's attitude (Dickens et al. 2019). Further, the structure and content of the BA concept could offer advantages and be of value for staff working with SFI in outpatient psychiatric care, as the intervention contributes to a more positive attitude among staff towards patients with BPD (Lindkvist et al. 2019; Lindgren et al. 2024; Eckerström et al. 2019). BA is based on a person‐centred approach and respect for the patient's autonomy, which has been shown to result in an equal interpersonal relationship between the patient and the nurse, where the focus shifts from a focus on symptoms towards a focus on the individual's current needs to cope with daily life (Arnold, Wärdig, and Hultsjö 2022; Enoksson et al. 2022). From the perspective of staff (Eckerström et al. 2019; Lindkvist et al. 2021), patients (Enoksson et al. 2022), and relatives (Lindkvist et al. 2024; Hultsjö, Rosenlund et al. 2023; Hultsjö, Appelfeldt et al. 2023), a person‐centred approach contributes to trust and confidence among all parties. Training in general is helpful in improving staff attitudes, especially where it is co‐delivered by experts by experience (Lamph et al. 2023, 2022). Thus, it could be constructive and helpful in SFI in outpatient mental health care if training draws upon the expertise of people with lived experience, in cooperation with employers and academics.

In the results, nurses also mentioned the challenge of connecting with elderly patients. There is a lack of interventions targeting this patient group, especially in terms of psychotherapy options. Multiprofessional outpatient interventions have been shown to be effective for depression in the elderly (Hölzel, Härter, and Hüll 2017). Furthermore, there is evidence that CBT, problem‐solving therapy, and life review therapy can help older adults gain insight into and improve their mental health (Hautzinger et al. 2017). Training in SFI can increase nurses' perceived competence, resulting in more effective suicide risk follow‐up (Solin, Tamminen, and Partonen 2021). This training needs to be regular to maintain improvements over time. This study provides a basis for considering the possibility of offering training in SFI tailored to patients with BPD and the elderly.

The ability to openly discuss patients with challenging conditions with colleagues emerged as important. Mental health nurses generally experience greater success in their work when there is good collaboration with colleagues (Bushell et al. 2021). Up to 95% of nurses find it helpful to discuss patients at risk of suicide with colleagues (McCann et al. 2013). Following completed suicides in inpatient mental health care, informal support from colleagues, where they could relate to each other's experiences, was perceived as more significant than the support offered by the employer (Alhamidi and Alyousef, 2022). Among the nurses in this study, challenges were found in being alone in SFI, and in difficult cases, they wanted the doctor to make the assessment. Mental health nurses may feel a significant responsibility to assess suicide risk themselves even though the doctor has the formal responsibility because these assessments may be considered complex and difficult (Derblom et al. 2021). Simply knowing that SFI is difficult and challenging can reduce the fear of making mistakes (Omerov et al. 2020). Nurses in this study expressed a desire to have a co‐evaluator in difficult cases to confirm that their assessments were correct. This is in line with nurses in outpatient mental health care in Swedish rural areas who have expressed a desire to organise a team around SFI where colleagues can share experiences and participate in each other's suicide risk assessments (Jansson and Graneheim 2018). Nurses in team‐based SFI were considered best suited to identify subtle changes in patient behaviour that could increase suicide risk, while other professional roles were considered better suited to perform the actual suicide risk assessment (Wittink et al. 2020).

Nurses in this study considered themselves competent to conduct SFI, but they regularly felt vulnerable and alone in suicide risk assessments as they lacked support or confirmation from colleagues, which left them exposed and vulnerable. Having exclusive responsibility for assessing suicide risk can heighten an individual's emotional vulnerability and moral distress (Jansson and Graneheim 2018). When suicide is perceived as avoidable, it can foster a culture of blame where the responsibility is placed on the nurse who works closest to the patient, which may lead the nurse to impose self‐blame and unrealistic demands on themselves (Robertson et al. 2010). Furthermore, this can evoke negative emotions of frustration, guilt, helplessness, anger, sadness, and mental health difficulties (Croft et al. 2023). This can in turn lead to feelings of incompetence and burnout that can be long‐lasting for some (Veilleux 2011). As suicide is not inevitable and can be prevented by asking considerate questions and encouraging help‐seeking behaviours regarding suicidal thoughts and actions (Nice 2018), it is important for nurses to have supportive colleagues who can alleviate their feelings and provide encouragement, comfort, and hope. This support enables nurses to persist in their work and meet their patients without fear of criticism (Jansson and Graneheim 2018). The results of this study support the importance of adequate education for nurses to create sufficient confidence to be able to talk about suicide and to assist patients in sharing their stories (Barker and Buchanan‐Barker 2010). Good clinical experience and personal competence, in harmony with teamwork and support from various professionals, provide increased confidence and patient safety in suicide risk follow‐up work (Bolster et al. 2015). There may be justification for evaluating the possibility of working more collaboratively around suicide risk follow‐up in outpatient mental health care. The lack of routines and structure regarding the handling of suicidal patients creates a sense of inadequacy (Rebair and Hulatt 2017).

In this study, the nurses stated that even though structured plans and risk assessment routines may be easy to understand, the content of the follow‐up conversations was somewhat unclear. The content of the follow‐up conversations is important as nurses emphasised different aspects of conversations with suicidal patients depending on whether their task was to assess suicide risk or to get to know the patient and build a caring relationship (Vandewalle et al. 2019). While nurses perceived structured plans, risk assessments, and routines as important, clinical experience was also highlighted as crucial in the assessments. Nurses caring for suicidal patients need to possess clinical experience for SFI to be effective (Talseth and Gilje 2011). Even family members can provide important information in suicide risk assessments as they possess information and often have a good insight into the patient's well‐being (Gorman et al. 2023) and can communicate about the potential risk of self‐harm, which is important for developing clear directions for monitoring the risk of suicide (Manuel et al. 2018). Contact with relatives in inpatient mental health care significantly increases the likelihood that the patient will contact outpatient mental health care services after discharge (Haselden et al. 2019). In this study, the nurses did not mention involving the patient's family in SFI, so there is room to review routines for how family members can be more actively involved and supported in SFI.

### 8.1 Limitations

One challenge associated with conventional content analyses is the risk of failing to develop a comprehensive understanding of the context, which may result in overlooking key categories. This can lead to findings that inaccurately represent the data. To establish credibility, all authors have been involved in the analysis process, and categories have been established by finding consensus among the authors regarding their meanings (Hsieh and Shannon 2005). Revealing experiences of nursing with suicidal patients can evoke feelings of not having done enough. To minimise the risk of social desirability bias (Bergen and Labonté 2020), which refers to respondents answering questions in a manner that is socially acceptable at the expense of their true opinions, none of the participants were known to the interviewers. It was ensured that both positive and negative statements were considered, thus enhancing the credibility of the interviews. Furthermore, time was allocated for reflections between interviewers and participants after each interview to ensure that no one left the room with unpleasant feelings. None of the participants expressed any concerns following the interview.

In qualitative research, the richness of data holds greater significance than the number of interviews conducted. The data demonstrated richness in content, and during the final interviews, it became evident that no additional data had emerged, thereby bolstering the credibility (Patton 2015). The authors' preconceptions risk affecting the results of a study, and this is a commonly occurring phenomenon (Polit and Beck 2021). To limit this risk, all authors have reflected on their own preconceptions and discussed with each other with the aim of preventing the preconceptions from affecting the results. To aid readers in assessing the transferability between the studied context and the context to which the results may be applied, a detailed portrayal of the demographic characteristics of the participants and method is provided (Patton 2015).

## 9 Conclusion & Clinical Implications for Practice

This study demonstrates that trust is a significant component of effective SFI. Nurses feel that they are competent and can make a difference in this work based on their profession but encounter challenges in following up on certain patients with challenging conditions. Offering both general training in suicide prevention and targeted training for patients with BPD and for the elderly can contribute to increased competence and more effective suicide follow‐ups. There can be advantages if the training is structured and incorporates concepts proven to be effective in other interventions, preferably drawing upon the expertise of people with lived experience, in cooperation with employers and academics.

Support from various professionals increases the nurses' sense of security as well as patient safety. Psychiatric facilities may therefore benefit from working more collaboratively, particularly in SFI, as this involves complex and difficult assessments. In team‐based work, recent graduates are given the opportunity to develop routines and clinical experience, of importance in SFI, to secure patient safety.

## Conflicts of Interest

The authors declare no conflicts of interest.

## Data Availability

Data available only on request due to privacy/ethical restrictions.
